# Clinical features and treatment of patients with esophageal cancer and a history of gastrectomy: a multicenter, questionnaire survey in Kyushu, Japan

**DOI:** 10.1111/dote.12439

**Published:** 2015-11-06

**Authors:** H. Okumura, N. Mori, T. Tanaka, M. Morita, Y. Toh, H. Saeki, Y. Maehara, K. Nakamura, H. Honda, N. Yoshida, H. Baba, S. Natsugoe

**Affiliations:** ^1^ Department of Digestive Surgery, Breast and Thyroid SurgeryKagoshima UniversityKagoshima; ^2^ Department of SurgeryKurume University School of MedicineFukuokaJapan; ^3^ Department of Gastroenterological SurgeryNational Hospital Organization Kyushu Cancer CenterFukuokaJapan; ^4^ Department of Surgery and ScienceKyushu UniversityFukuokaJapan; ^5^ Department of Clinical RadiologyKyushu UniversityFukuokaJapan; ^6^ Department of Gastroenterological SurgeryKumamoto UniversityKumamotoJapan

**Keywords:** chemoradiation, esophageal cancer, esophagectomy, gastrectomy, prognosis

## Abstract

It is still controversial whether patients with a history of gastrectomy have high risk of esophageal carcinogenesis. On the other hand, the treatment strategy for esophageal cancer patients after gastrectomy is complicated. The association between histories of gastrectomy and esophageal carcinogenesis was retrospectively analyzed, and the treatment of esophageal cancer patients after gastrectomy was evaluated based on questionnaire data collected from multiple centers in Kyushu, Japan. The initial subject population comprised 205 esophageal cancer patients after gastrectomy. Among them, 108 patients underwent curative surgical treatment, and 70 patients underwent chemoradiation therapy (CRT). The time between gastrectomy and esophageal cancer development was longer in peptic ulcer patients (28.3 years) than in gastric cancer patients (9.6 years). There were no differences in the location of esophageal cancer according to the gastrectomy reconstruction method. There were no significant differences in the clinical background characteristics between patients with and without a history of gastrectomy. Among the 108 patients in the surgery group, the 5‐year overall survival rates for stages I (*n* = 30), II (*n* = 18), and III (*n* = 60) were 68.2%, 62.9%, and 32.1%, respectively. In the CRT group, the 5‐year overall survival rate of stage I (*n* = 29) was 82.6%, but there were no 5‐year survivors in other stages. The 5‐year overall survival rate of patients with CR (*n* = 33) or salvage surgery (*n* = 10) was 61.2% or 36%, respectively. For the treatment of gastrectomized esophageal cancer patients, surgery or CRT is recommended for stage I, and surgery with or without adjuvant therapy is the main central treatment in advanced stages, with surgery for stage II, neoadjuvant therapy + surgery for stage III, and CRT + salvage surgery for any stage, if the patient's condition permits.

## Introduction

Gastrectomy for peptic ulcer and cancer is the major abdominal surgery in Japan. Recently, the prognosis of patients with gastric cancer has improved with early detection and advances in perioperative management. Therefore, esophageal cancer is discovered in patients with a history of gastrectomy due to both gastroduodenal peptic ulcer and metachronous gastric cancer.^1^ Biologically, the carcinogenesis of esophageal cancer is related to gastroesophageal reflux of bile acid.^2^, ^3^ Although there are some clinical reports evaluating whether gastrectomy is associated with esophageal carcinogenesis, the number of patients was relatively few, ranging from 11 to 72.^4^, ^5^, ^6^, ^7^, ^8^, ^9^, ^10^ However, it is still controversial whether gastrectomized patients have a high risk of esophageal carcinogenesis, and thus analysis involving a large number of patients is warranted.

Regarding the surgical treatment for esophageal cancer after gastrectomy, colon or jejunum is used for reconstruction instead of a gastric tube. Such methods tend to lead to higher morbidity and mortality.^11^ Recently, chemoradiation therapy (CRT) was found to be one of the most useful treatments for esophageal cancer.^12^, ^13^ Thus, the treatment of gastrectomized esophageal cancer patients should be seriously discussed on the basis of patients' benefit.

In this study, clinicopathological data of gastrectomized esophageal cancer patients were collected from multiple institutes in Kyushu, Japan, the relationship between gastrectomy and esophageal carcinogenesis was retrospectively analyzed, and the treatments were evaluated.

## Patients and Methods

### Patients

The initial subject population comprised 205 esophageal cancer patients after gastrectomy. Their data were collected from 12 departments in 9 university hospitals and one cancer center with which the members of the Kyushu study group for adjuvant therapy of esophageal cancer were associated. The study period ranged from 1998 to 2014. There were 200 men and 5 women with an average age of 68.1 years. Among them, 174 patients had undergone distal gastrectomy, and 31 patients had undergone total gastrectomy due to either gastroduodenal peptic ulcer or gastric cancer. Reconstruction after gastrectomy was performed by Billroth‐I (B‐I) in 137 patients, Billroth‐II (B‐II) in 30, and Roux‐en‐Y (R‐Y) in 38 (Table 1). Clinicopathological data were retrospectively evaluated based on the tumor node metastasis (TNM) classification of the International Union against Cancer^14^ and the Japanese Classification of Esophageal Cancer.^15^, ^16^ Complications were evaluated according to the specific classification for esophageal surgery.^17^ The median follow‐up period of this cohort was 27 months (range 1–140 months).

**Table 1:** Clinicopathological characteristics according to gastric cancer and peptic ulcer patients

Clinical variables	Total Cases	*Gastric cancer	*Peptic ulcer	* *P*‐value
	*n* = 205	*n* = 118	*n* = 87	
Age (mean ± SD)	68.1 ± 7.9	69.1 ± 8.1	66.7 ± 7.5	0.03
Gender				0.3
Male	200 (97.6%)	114 (96.6%)	86 (98.8%)	
Female	5 (2.4%)	4 (3.4%)	1 (1.2%)	
Methods of gastrectomy				0.0001
Distal gastrectomy	174 (84.9%)	90 (76.3%)	84 (96.6%)	
Total gastrectomy	31 (15.1%)	28 (23.7%)	3 (3.4%)	
Methods of reconstruction				0.0001
B‐I	137 (66.8%)	75 (63.6%)	62 (71.3%)	
B‐II	30 (14.6%)	8 (6.8%)	22 (18.6%)	
R‐Y	38 (18.5%)	35 (29.7%)	3 (2.5%)	
Duration from gastrectomy to esophageal cancer (mean years ± SD)	17.6 ± 12.8	9.6 ± 7.6	28.3 ± 10.3	0.0001
Histology				0.7
SCC	194 (94.6%)	113 (95.8%)	81 (93.1%)	
Adenocarcinoma	5 (2.4%)	2 (1.7%)	3 (3.4%)	
Other	6 (2.9%)	3 (2.5%)	3 (3.4%)	
Location				0.2
Upper	38 (18.5%)	19 (16.1%)	19 (21.8%)	
Middle	103 (50.2%)	66 (55.9%)	37 (42.5%)	
Lower	64 (31.2%)	33 (28.0%)	33 (37.9%)	
Tumor depth (cT)				0.1
T1	71 (34.6%)	49 (41.5%)	22 (25.3%)	
T2	29 (14.1%)	15 (12.7%)	14 (16.1%)	
T3	72 (35.1%)	38 (32.2%)	34 (39.1%)	
T4	33 (16.1%)	16 (13.6%)	17 (19.5%)	
Lymph node metastasis (cN)				0.07
N0	98 (47.8%)	65 (55.1%)	33 (37.9%)	
N1	46 (22.4%)	24 (20.3%)	22 (25.3%)	
N2	33 (16.1%)	14 (11.9%)	19 (21.8%)	
N3	28 (13.7%)	15 (12.7%)	13 (14.9%)	
Distant metastasis (cM)				0.9
M0	193 (94.1%)	111 (94.1%)	82 (94.3%)	
M1	12 (5.9%)	7 (5.9%)	5 (5.7%)	
cStage				0.03
I	73 (35.6%)	49 (41.5%)	24 (27.6%)	
II	31 (15.1%)	21 (17.8%)	10 (11.5%)	
III	89 (43.4%)	41 (34.7%)	48 (55.2%)	
IV	12 (5.9%)	7 (5.9%)	5 (5.7%)	

*P‐value was estimated between gastric cancer group and peptic ulcer group. B‐I: Billroth‐I, B‐II: Billroth‐II, R‐Y: Roux‐en‐Y, SCC: squamous cell carcinoma; SD, standard deviation.

### The clinical criteria for the response to CRT

The clinical criteria for the response of target lesions were as follows.^18^ Complete response (CR) was the disappearance of all target lesions, as well as secondary changes associated with the tumors. Partial response (PR) was at least a 30% decrease in the sum of the greatest dimensions of target lesions, taking as reference the baseline sum of the greatest dimensions. Progressive disease (PD) was at least a 20% increase in the sum of the greatest dimensions of the target lesions, taking as reference the smallest sum of greatest dimensions recorded since the treatment started. Stable disease (SD) was defined as neither PR nor PD.

### Statistical analysis

Statistical analysis of group differences was performed using the χ^2^ test and Student's *t*‐test. The Kaplan–Meier method was used for survival analysis, and differences in survival were evaluated using the log‐rank test. Multivariate analysis was performed using Cox‐hazard model analysis. The *P* values in this study were two sided, and a *P* value of <0.05 was considered significant. All statistical analyses were performed using the software package StatView version 5.0 (Abacus Concepts, Berkeley, CA, USA).

## Results

### Treatments of esophageal cancer patients

Among 205 patients, initial treatment methods for esophageal cancer were as follows: curative surgical treatment in 108 cases; CRT in 70 cases; endoscopic treatment in 13 cases; radiation therapy in 6 cases; chemotherapy in 2 cases; and best supportive care in 6 cases. Since there were few patients treated with therapies other than surgical or CRT treatment, these patients were excluded. Thus, 108 surgically treated cases (surgery group) and 70 CRT cases (CRT group) were actually evaluated (Table 2). In the surgery group, esophagectomy through right thoracotomy and left thoracotomy was performed in 81 and 11 patients, respectively; 6 underwent thoracoscopic resection, and 10 underwent transhiatal blunt resection. Neoadjuvant therapy was given to 11 patients with CRT and 18 patients with chemotherapy. Reconstruction was performed using colon interposition in 86 cases and jejunum in 22 cases, and their routes were posterior mediastinal in 19 cases, retrosternal in 4 cases, and subcutaneous in 85 cases.

**Table 2:** Clinicopathological characteristics according to treatment method for esophageal cancer

Clinical variables	Surgery group	CRT group	*P*‐value
	*n* = 108	*n* = 70	
Age (mean years ± S.D.)	66.9 ± 7.7	69.6 ± 7.9	0.02
Gender			0.3
Male	106 (98.1%)	67 (95.7%)	
Female	2 (1.9%)	3 (4.3%)	
Location of the tumor			0.1
Upper	18 (16.7%)	18 (25.7%)	
Middle	50 (46.3%)	36 (51.4%)	
Lower	40 (37.0%)	16 (22.9%)	
Histological type			0.9
SCC	103 (95.4%)	67 (95.7%)	
Adenocarcinoma	1 (0.9%)	1 (1.4%)	
Other	4 (3.7%)	2 (2.9%)	
Tumor depth (cT)			0.02
T1	28 (25.9%)	27 (38.6%)	
T2	19 (17.6%)	7 (10.0%)	
T3	48 (44.4%)	20 (28.6%)	
T4	13 (12.0%)	16 (22.9%)	
Lymph node metastasis (cN)			0.08
N0	43 (39.8%)	37 (52.9%)	
N1	32 (29.6%)	11 (15.7%)	
N2	20 (18.5%)	10 (14.3%)	
N3	13 (12.0%)	12 (17.1%)	
Distant metastasis (cM)			0.0003
M0	108 (100%)	62 (88.6%)	
M1	0 (0%)	8 (11.4%)	
cStage			0.0002
I	30 (27.8%)	29 (41.4%)	
II	18 (9.3%)	9 (12.9%)	
III	60 (55.6%)	24 (34.3%)	
IV	0 (0%)	8 (11.4%)	

In the CRT group, 65 patients were definitively irradiated with 50 Gy or more, while 5 patients received less than 50 Gy. Ten of 65 patients with definitive CRT underwent salvage surgery.

### Clinicopathological characteristics in gastric cancer and peptic ulcer patients

**Figure 1:**
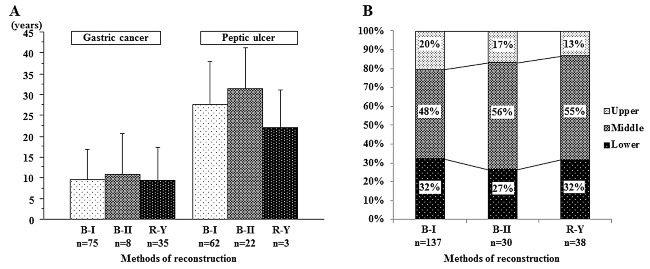
(A) Time from gastrectomy to diagnosis of esophageal cancer. There are no differences in time from gastrectomy to the diagnosis of esophageal cancer by the reconstruction method; the times are 9.6, 10.8, and 9.4 years in gastric cancer patients, and 27.5, 31.3, and 22.0 years in peptic ulcer patients for B‐I, B‐II, and R‐Y reconstructions, respectively. (B) Tumor location according to reconstruction method. There is no difference in tumor location by reconstruction method (B‐I, B‐II, and R‐Y).

Clinicopathological characteristics were compared between 118 gastric cancer and 87 gastroduodenal peptic ulcer patients (Table 1). The age of esophageal cancer diagnosis was lower in peptic ulcer patients (66.7 years) than in gastric cancer patients (69.1 years) (*P* = 0.03). Most patients (97.6%) were male, with no difference between the groups. As for the method of gastrectomy, distal gastrectomy was more frequent for peptic ulcer, and total gastrectomy was more frequent for gastric cancer (*P* = 0.0001). B‐I reconstruction was the main method, but B‐II reconstruction was more frequent for peptic ulcer, and R‐Y reconstruction was more frequent for gastric cancer (*P* = 0.0001). The time between gastrectomy and esophageal cancer discovery was longer in peptic ulcer patients than in gastric cancer patients (28.3 years, 9.6 years, respectively; *P* = 0.0001). There was no significant difference in this time according to reconstruction method (Fig. 1A). There were no differences in histology and location of esophageal cancer according to reconstruction method between peptic ulcer and gastric cancer patients (Fig. 1B). As for TNM classification, peptic ulcer patients tended to have more advanced lymph node metastases and significantly more advanced clinical stage (*P* = 0.03).

### Clinicopathological characteristics according to treatment method for esophageal cancer

The clinical signatures were compared between the surgery group (*n* = 108) and the CRT group (n = 70) (Table 2). Age was younger in the surgery group (66.9 years) than in the CRT group (69.6 years; p = 0.02). There were no differences in location and histology of esophageal cancer. As for TNM classification, the CRT group had more T1 or T4 tumors, more distant lymph node metastases, and more stage I or stage IV cases than the surgery group (*P* = 0.02, 0.0003, 0.0002, respectively).

### Clinical outcome of the surgery group

Postoperative complications were found in 51 of 108 patients (47.2%). Anastomotic leak, pulmonary complication, conduit necrosis, and mortality were found in 28 patients (26.0%), 23 patients (21.3%), 5 patients (4.6%), and 6 patients (5.6%), respectively. In this series, the mortality rate was 5.6% (6/108), and such postoperative complications were significantly associated with mortality (Table 3).

**Table 3:** Morbidity and mortality in the surgery group (*n* = 108)

Clinical variables	Mortality (*n* = 6)	*P*‐value
Anastomotic leak (Type II)		0.001
(−) (*n* = 80)	1 (1.3%)	
(+) (*n* = 28)	5 (17.9%)	
Pulmonary complication		0.005
(−) (*n* = 85)	2 (2.4%)	
(+) (*n* = 23)	4 (17.4%)	
Conduit necrosis (type III)		0.0006
(−) (*n* = 103)	4 (3.9%)	
(+) (*n* = 5)	2 (40.0%)	

Anastomotic leak (Type II): localized defect requiring interventional but not surgical therapy, for example, interventional radiology drain, stent, or bedside opening and packing of incision.

Conduit necrosis (Type III): conduit necrosis extensive, Treatment – treated with conduit resection with diversion.^17^

Comparing morbidity or mortality and surgical treatment methods, there were no significant differences between morbidity or mortality and surgical approach, neoadjuvant therapy, reconstruction organ, and reconstruction route, although patients who received neoadjuvant therapy tended to have pulmonary complications (*P* = 0.09) (Table 4).

**Table 4:** Comparisons of morbidity, mortality, and operation method in the surgery group (n = 108)

Clinical variables	Anastomotic leak (Type II) (*n* = 28)	*P*	Pulmonary complication (*n* = 23)	*P*	Conduit necrosis (type III) (*n* = 5)	*P*	Mortality (*n* = 6)	*P*
Surgical approach		0.6		0.7		0.7		0.7
Rt. Thoracotomy(*n* = 81)	22 (27.2%)		18 (22.2%)		4 (4.9%)		5 (6.2%)	
Thoracoscopic (*n* = 6)	2 (33.3%)		2 (33.3%)		0 (0%)		0 (0%)	
Blunt (*n* = 10)	3 (30.0%)		2 (20.0%)		1 (10.0%)		1 (10.0%)	
Lt. thoracotomy (*n* = 11)	1 (9.0%)		1 (9.0%)		0 (0%)		0 (0%)	
Neoadjuvant therapy		0.8		0.09		0.5		0.2
+ (*n* = 29)	8 (27.6%)		9 (31.0%)		2 (6.9%)		3 (10.3%)	
− (*n* = 79)	20 (25.3%)		14 (17.7%)		3 (3.8%)		3 (3.8%)	
Reconstruction organ		0.1		0.9		1.0		0.2
Colon (*n* = 86)	25 (29.1%)		18 (20.9%)		4 (4.7%)		6 (7.0%)	
Jejunum (*n* = 22)	3 (13.6%)		5 (22.7%)		1 (4.5%)		0 (0%)	
Reconstruction route		0.5		0.4		0.9		0.5
Post. med. (*n* = 19)	4 (21.1%)		4 (21.1%)		1 (5.3%)		2 (10.5%)	
Retrosternal (*n* = 4)	2 (50.0%)		2 (50.0%)		0 (0%)		0 (0%)	
Subcutaneous (*n* = 85)	22 (25.9%)		17 (20.0%)		4 (4.7%)		4 (4.7%)	

Rt.: right, Lt.: left, Post. Med.: posterior mediastinum. Anastomotic leak (Type II): localized defect requiring interventional but not surgical therapy, for example, interventional radiology drain, stent or bedside opening and packing of incision. Conduit necrosis (Type III): conduit necrosis extensive, Treatment – treated with conduit resection with diversion.^17^

Thirty patients (27.8%) had relapse of disease in the Surgery group, and the mode of initial recurrence was lymph node (*n* = 16), hematogenous (*n* = 11), and local (*n* = 3).

**Figure 2:**
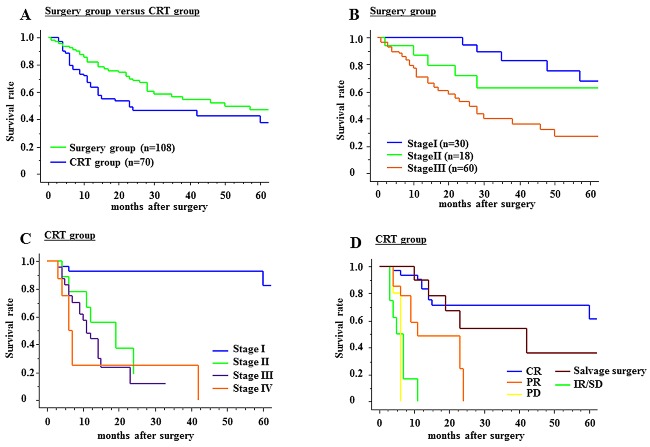
Survival analyses. (A) The 3‐year and 5‐year cumulative survival rates of the surgery group are 57.2% and 45.6%, while those of the chemoradiation therapy (CRT) group are 46.3% and 38.0%. The 5‐year survival rate is significantly better in the surgery group than in the CRT group (*P* = 0.02). (B) The 5‐year overall survival rates of the surgery group for stage I (*n* = 30), II (*n* = 18), and III (*n* = 60) are 68.2%, 62.9%, and 32.1%, respectively (*P* = 0.0003). (C) The 5‐year overall survival rate of the CRT group for stage I (*n* = 29) is 82.6%, but there are no survivors at 5 years in stages II (*n* = 9), III (*n* = 24), and IV (*n* = 8) in the CRT group. (D) Estimating the survival rate according to clinical response including salvage surgery in the CRT group, the 5‐year survival rate of CR (*n* = 33) is 61.2%, and that of salvage surgery patients (*n* = 10) is 36%, with no survivors at 5 years in the partial response (PR; *n* = 14), stable disease (SD; *n* = 8), and progressive disease (PD; *n* = 5) groups.

The 3‐year and 5‐year cumulative survival rates of the surgery group were 57.2% and 45.6% (Fig. 2A); for stage I (*n* = 30), II (*n* = 18), and III (*n* = 60) the 5‐year cumulative survival rates were 68.2%, 62.9%, and 32.1%, respectively (Fig. 2B), and the 5‐year disease‐specific survival rates were 84.4%, 68.7%, and 35.5%, respectively (data not shown).

### Clinical outcomes of the CRT group

For clinical responses after CRT, the numbers of patients with CR, PR, SD, and PD were 40, 16, 8, and 6, respectively (Table 5). According to clinical stage, the response to CRT was significantly better in patients with early‐stage disease (*P* = 0.0001) (Table 5). Five patients whose radiation dose was less than 50 Gy had significantly worse clinical responses. Ten patients underwent salvage surgery, one (10%) of whom died. The distribution of salvage surgery had no significant correlation with clinical response (Table 5).

**Table 5:** Analyses of clinical response in the chemoradiation therapy (CRT) group

Clinical variables	Clinical response	*P*‐value
	CR/PR/SD/PD	
	*n* = 40/16/ 8/ 6	
Clinical stage		0.0001
I (*n* = 29)	26/2/1/0	
II (*n* = 9)	3/4/ /2	
III (*n* = 24)	10/8/5/1	
IV (*n* = 8)	1/2/2/3	
Irradiation dose		0.002
50 Gy ≤ (*n* = 65)	40/15/5/5	
50 Gy > (*n* = 5)	0/1/3/1	
Salvage surgery		0.6
(+) (*n* = 10)	7/2/0/1	
(−) (*n* = 60)	33/14/8/5	

CR, complete response; PD, progressive disease; PR, partial response; SD, stable disease.

The 3‐year and 5‐year cumulative survival rates of the CRT group were 46.3% and 38.0%, respectively, which were significantly worse than the surgery group (*P* = 0.02, Fig. 2A). The 5‐year overall survival rate of stage I (*n* = 29) was 82.6%, with a disease‐specific survival rate of 88.9% (data not shown), which was not significantly different from the surgery group, but there were no 5‐year survivors in stages II (*n* = 9), III (*n* = 24), and IV (*n* = 8) in the CRT group. In these stage groupings, the prognosis was significantly worse than in the surgery group (Fig. 2C, *P* = 0.0001). Estimating the survival ratio according to clinical response including salvage surgery, the 5‐year survival rate of CR (*n* = 33) was 61.2% and that of salvage surgery patients (*n* = 10) was 36.0%, but there were no 5‐year survivors with PR (*n* = 14), SD (*n* = 8), and PD (*n* = 5) (*P* = 0.0001, Fig. 2D).

### Analysis of prognostic factors

On univariate analysis, sex, disease for which gastrectomy was performed, method of gastrectomy, reconstruction method, and location of esophageal cancer were not prognostic factors, but treatment for esophageal cancer (*P* = 0.02), cT, and cN (*P* = 0.0001, each) were prognostic factors. On multivariate analysis, treatment for esophageal cancer, cT, and cN were the independent prognostic factors (*P* = 0.0001, 0.006, and 0.001, respectively, Table 6).

**Table 6:** Analyses of prognostic factors

	Univariate			Multivariate		
Clinical variables	HR	95% CI	P	HR	95% CI	*P*
Gender						
Male	1	0.45–2.3	0.26			
Female	0.32					
Disease of gastrectomy						
Peptic ulcer	1	0.55–1.3	0.48			
Gastric cancer	0.85					
Method of gastrectomy						
Distal gastrectomy	1	0.50–2.2	0.88			
Total gastrectomy	1.1					
Reconstruction method						
B‐I	1.3	0.65–2.5	0.47			
B‐II	1.4	0.65–3.2	0.38			
R‐Y	1					
Location of esophageal cancer						
Upper	1	0.49–1.6	0.85			
Middle	0.88	0.57–2.0	0.67			
Lower	1.1					
Treatment of esophageal cancer						
Surgery	1	1.1–2.7	0.02	1	1.7–4.3	0.0001
CRT	1.7			2.7		
cT						0.006
cT1	1	1.9–6.5	0.0001	1	1.3–5.8	
cT2‐4	3.5			2.8		
cN						0.001
cN0	1	2.2–5.8	0.0001	1	1.5–4.8	
cN1‐3	3.5			2.7		

CI, confidence intervals; CRT, chemoradiation therapy; HR, hazard ratio.

## Discussion

Some reports have attempted to determine esophageal carcinogenesis based on several aspects, including its association with a history of gastrectomy.^2^, ^3^, ^4^, ^5^, ^6^, ^7^, ^8^, ^9^, ^10^, ^19^, ^20^, ^21^ In general, the incidence of gastrectomy in Japanese was 0.87%.^7^ On the other hand, esophageal cancer after gastrectomy occurred in the range from 4.4–10.4%, with an obviously higher incidence than in the general population.^4^, ^5^, ^6^, ^7^, ^8^, ^9^, ^10^ In basic research using an animal reflux model, there is significant evidence that gastroesophageal reflux induces both squamous cell carcinoma and adenocarcinoma of the esophagus that depends on the strength of the reflux effect.^3^ In a knockout mice model, additive genetic changes and carcinogen effect working together increased the incidence of forestomach and esophageal cancer, indicating the necessity of host and environmental factors for esophageal carcinogenesis.^19^ On pathological estimation, 20% of gastrectomized healthy patients had reflux disease and esophagitis, and 40% of patients had dysplasia in the lower esophagus.^20^ In biological molecular assessment, positive p53 expression was found in not only the primary tumor, but also intraepithelial neoplasia around the tumor in gastrectomized esophageal cancer patients, indicating that chronic reflux after gastrectomy may be related to carcinogenesis in addition to environmental and genetic factors.^21^ In clinical analyses, some authors supported a correlation between post‐gastrectomy status and incidence of esophageal cancer,^9^, ^10^ while others did not.^5^, ^6^, ^8^ The lower esophagus (40.3%) was reportedly the most frequent location to be affected by tumor.^9^ In this series, the proportions of upper, middle, and lower esophageal cancers were 18.5%, 50.2%, and 31.2%, respectively, almost the same as the data from the comprehensive registry of esophageal cancer in Japan 2006 (13.4%, 48.7%, and 31.6%, respectively).^22^ Moreover there was no difference between tumor location and reconstruction methods, either (Fig. 1B). The duration between gastrectomy and esophageal cancer development was reportedly longer in the peptic ulcer group (13.4–28.9 years) than in the gastric cancer group (5.8–11.5 years), which was in accordance with the present data (28.3 years in the peptic ulcer group and 9.6 years in the gastric cancer group, Table 1).^4^, ^5^, ^6^, ^7^, ^8^, ^9^, ^10^ However, no significant difference was found in the interval between gastrectomy and esophageal cancer discovery in both groups according to reconstruction method (Fig. 1A). Furthermore, the mean age of patients was 68.1 years in the present study, which is almost the same as that reported in the comprehensive registry of esophageal cancer in Japan; the percentages of tumor stages I, II, III, and IV were 35.6%, 15.1%, 43.45%, and 5.9%, respectively, in the present cohort and 27.2%, 22.6%, 28.8%, and 13.5%, respectively, in the data from comprehensive registry.^22^ The percentage of stage I or II was almost 50% in both datasets, which did not indicate any obvious difference between these two groups. Taken together, there were no significant differences in the clinical background characteristics between patients with and without a history of gastrectomy, although reflux disease may be related to carcinogenesis.

The duration between gastrectomy and esophageal cancer discovery was significantly longer in peptic ulcer patients than in gastric cancer patients, and peptic ulcer patients tended to have more advanced lymph node metastases and a significantly more advanced stage. The reason for this phenomenon was patients who underwent gastrectomy for gastric cancer were well followed up. However, patients who underwent gastrectomy for peptic ulcer were poorly followed up because most of them stopped going to hospital during their long follow‐up period. Therefore, they tended to have more advanced stage due to fewer opportunities to find the tumors.

Considering treatment strategy, it is usual to evaluate surgical treatment, multimodal treatment, and definitive CRT to determine which is better. In the surgery group, the mortality rate was 5.6%, which is not different from the data of the national survey in Japan 2005, at 4.9%.^23^ The cumulative 5‐year survival rates for stages I and II were acceptable (overall 68.2%, 62.9%, and disease specific 84.4%, 68.7%, respectively), but the prognosis of stage III had room for improvement (overall 32.1% and disease specific 35.5%). Among 30 stage I patients, 7 died, due to pneumonia in 4, recurrence of cancer in 2, and heart failure in 1. Most of the causes of death were nonrecurrent disease, and 10 cases were censored within 3 years, which made overall survival worse. Kato *et al*. reported that the 5‐year survival rate of 50 gastrectomized patients was 35.9%, and Wada *et al*. reported that the cause‐specific 5‐year survival rate of 72 patients was 65%, which was better compared with non‐gastrectomized patients.^6^, ^9^ Thus, we are able to expect better survival for gastrectomized esophageal patients despite the stressful and complicated procedure.^6^, ^8^, ^9^ However, to improve the clinical outcome for stage III patients, neoadjuvant therapy may be considered.

On the other hand, in the CRT group, overall survival of stage I (82.6%) was acceptable, but patients with stage II–IV had no survival benefit. Similarly, although the 5‐year survival rate of patients with CR (61.2%) was acceptable, patients with PR, SD, and PD did not have acceptable outcomes because they were all dead within 5 years. In these patients, 10 patients underwent salvage surgery. Since their 5‐year survival rate was 36%, this treatment is worth trying. The most common reason for using CRT for gastrectomized patients with advanced esophageal cancer is their poor systemic condition. Aiko *et al*. reported that lymph node metastases tended to occur within the thoracic region for gastrectomized esophageal cancer patients because of altered lymphatic flow due to gastrectomy. Thus, we might be able to limit the irradiation field in performing CRT for these patients considering their condition.^8^

Finally, cT, cN, and initial treatment method such as surgery or CRT were the independent prognostic factors. This result was thought to be reasonable, and selection of the initial treatment based on the patients' condition and tumor extension is essential.

The present study had the following limitations. This was a retrospective, multicenter study with some patient selection bias. Even though the sample size of this study was large, it was still small for some specific subgroup analyses, such as comparisons of morbidity, mortality, and operation method in the surgery group and analyses of clinical response in the CRT group. There were also institutional biases and historical changes in the preoperative diagnosis, neoadjuvant therapy, and postoperative management. Therefore, these results should be understood in light of the above limitations.

In conclusion, there were no significant differences in the clinical background characteristics between patients with and without a history of gastrectomy. For the treatment of gastrectomized esophageal cancer patients, surgery or CRT is recommended for stage I, and surgery with or without adjuvant therapy is the main central treatment in advanced stage, with surgery for stage II, neoadjuvant + surgery for stage III, and CRT + salvage surgery for any stage, if the patient's condition permits.
